# Critical Care Medical Centers May Play an Important Role in Reducing the Risk of COVID-19 Death in Japan

**DOI:** 10.1007/s42399-020-00547-y

**Published:** 2020-09-29

**Authors:** Yohei Ishikawa, Toru Hifumi, Mitsuyoshi Urashima

**Affiliations:** 1 Department of Emergency and Critical Care Medicine, St. Luke’s International Hospital, 9-1 Akashi-cho, Chuo-ku, Tokyo, 104-8560 Japan; 2 Division of Molecular Epidemiology, The Jikei University School of Medicine, 3-19-18, Nishi-shinbashi, Minato-ku, Tokyo, 105-8471 Japan

**Keywords:** COVID-19, SARS-CoV-2, Mortality, Polymerase chain reaction

## Abstract

Marked differences in COVID-19 mortalities have been observed among 47 prefectures in Japan. Here, we explored associations between COVID-19 mortalities and medical and public health capacities in individual prefectures. The following data by prefecture were abstracted from open resources provided by the Ministry of Health, Labour and Welfare in Japan as of May 24, 2020: total number of COVID-19 deaths; polymerase chain reaction (PCR)-positive ratio (i.e., number of patients with PCR-positive results/number of patients aiming diagnosis of COVID-19 or individuals in close contacted with COVID-19 patients); number of call centers, outpatient centers, and hospital beds specifically for patients diagnosed with COVID-19; and others. The primary outcome was COVID-19 mortality per million population. Multiple and simple linear regression models were applied. Two variables were significantly associated with COVID-19 mortality: PCR-positive ratio (*P* < 0.001) and number of critical care medical centers per million population (*P* = 0.001). PCR-positive ratio was positively associated with COVID-19 mortality (aR-sqr = 0.522). Low PCR-positive ratio, especially ≤ 4%, was associated with low COVID-19 mortality. Critical care medical centers may also play an important role in reducing the risk of COVID-19 death.

## Introduction

The coronavirus disease 2019 (COVID-19) pandemic resulted in 337,687 deaths around the world [1] and 830 deaths in Japan consisting of 47 prefectures, as of May 24, 2020 [2]. Marked differences in COVID-19 mortalities have been observed among 47 prefectures in Japan. For example, mortalities have exceeded 20 per million population in Ishikawa, Toyama, and Tokyo, compared to less than 1 per million population in 24 other prefectures. Moreover, huge disparities exist in medical and public heath capacities per capita across the 47 prefectures. We therefore explored associations between COVID-19 mortalities and medical and public health capacities (e.g., hospital beds per population) in individual prefectures as an ecological study.

## Methods

The following data by prefecture were abstracted from open resources provided by the Ministry of Health, Labour and Welfare in Japan as of May 24, 2020 [2, 3]: total number of COVID-19 deaths; polymerase chain reaction (PCR)-positive ratio (i.e., number of patients with PCR-positive results/number of patients aiming diagnosis of COVID-19 or individuals in close contacted with COVID-19 patients); number of call centers, outpatient centers, and hospital beds specifically for patients diagnosed with COVID-19; and others. Moreover, minutes of the ambulance call to arriving at hospital was obtained from the Ministry of Public Management, Home Affairs, Posts and Telecommunications [4]. Four potential confounders were used for adjustment: population density (/km^2^), percentage of the population ≥ 65 years old, university education (%), and mean income. The primary outcome was COVID-19 mortality per million population. Multiple and simple linear regression models were applied to screen and confirm significant parameters, respectively. Values showing two-sided *P* values < 0.05 were considered statistically significant, and each model was evaluated by adjusted R-squared (aR-sqr). Data were analyzed using Stata version 14.0 software (StataCorp LP, College Station, TX). Institutional review board approval was not sought, due to the use of publicly available, de-identified data, per the usual institutional policy.

## Results

**Fig. 1:**
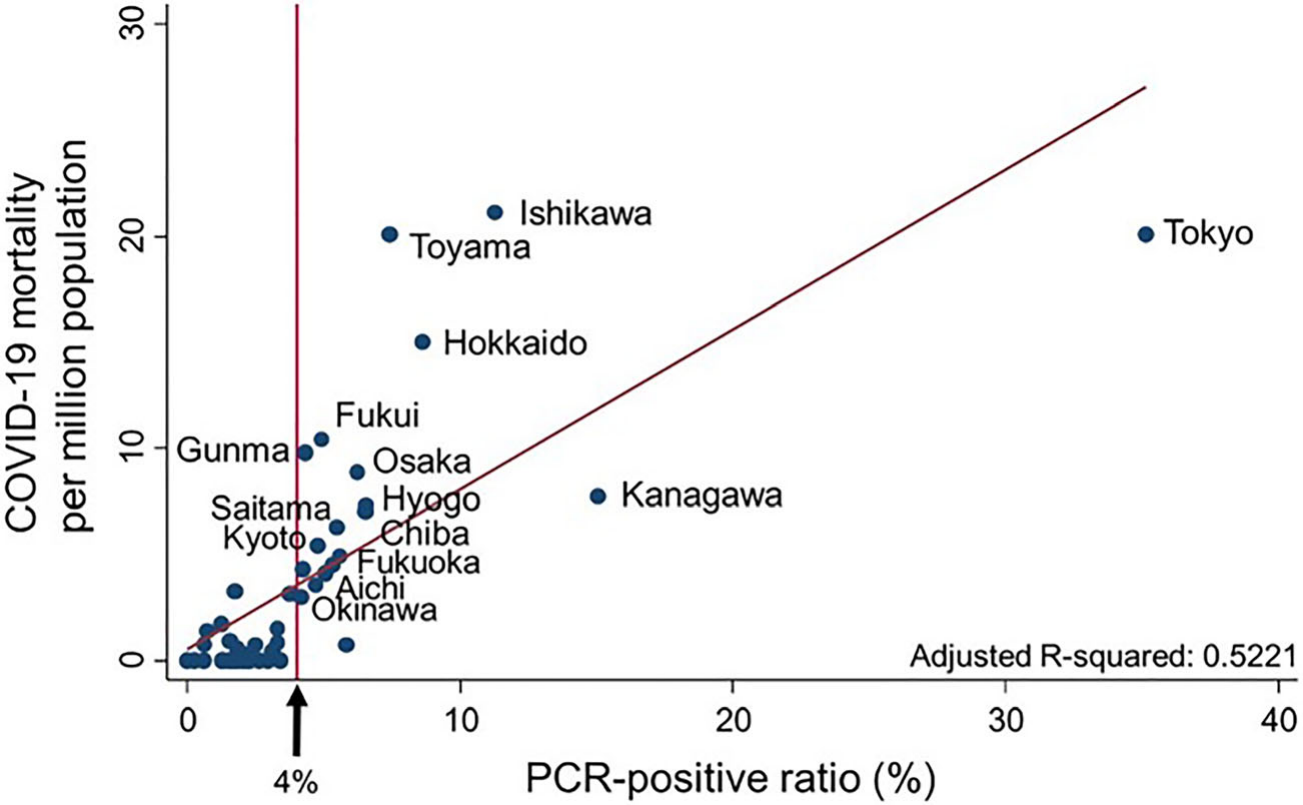
Scatter plot of COVID-19 mortality and PCR-positive ratio (%)

**Fig. 2:**
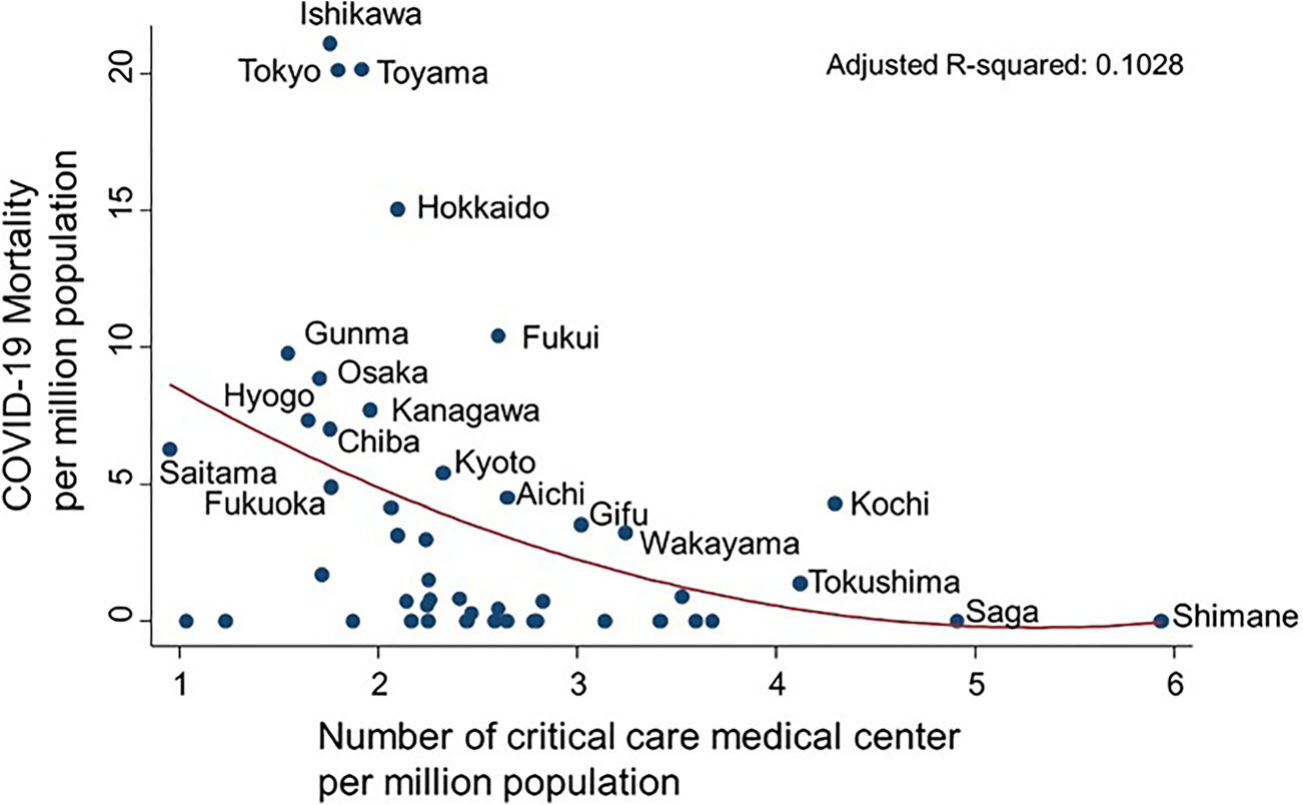
Number of critical care medical centers per million population

Multivariate analysis was performed using 14 parameters of medical and public health capacities (Table 1). The aR-sqr of this model was 0.679. Two variables were significantly associated with COVID-19 mortality: PCR-positive ratio (*P* < 0.001) and number of critical care medical centers per million population (*P* = 0.001). Scatter plots were drawn for both of these variables. PCR-positive ratio ranged from 35% of 14,653 PCR analyses performed in Tokyo to 0% of 602 analyses performed in Iwate and was positively associated with COVID-19 mortality (aR-sqr = 0.522) (Fig. 1). Median mortality was 0.00 (interquartile range (IQR), 0.00–0.78) in prefectures with ≤ 4% PCR**-**positivity (*n* = 28), which was significantly less than 7.03 (IQR, 4.30–10.42) in those with PCR-positivity > 4% (*n* = 19; Mann-Whitney test, *P* < 0.0001). Moreover, number of critical care medical centers ranged from 5.93 per million population in Shimane, where COVID-19 mortality was zero, to 0.95 per million population in Saitama, and was negatively associated with COVID-19 mortality (aR-sqr = 0.1028) (Fig. 2).

**Table 1:** A multiple linear regression model of COVID-19 mortality

	Median (IQR)	Coefficiency	95% CI	*P* value
Capacities specific for COVID-19				
PCR positive (%)	3.1 (1.7–5.3)	0.988	0.551 to 1.425	< 0.001
Call center per million (no.)	5.4 (3.2–7.4)	− 0.118	− 0.684 to 0.449	0.67
Outpatient center per million (no.)	14.2 (11.2–20.0)	0.058	− 0.266 to 0.382	0.72
Hospital beds per million (no.)	117 (80–178)	0.009	− 0.002 to 0.209	0.12
Community health in general				
Number of institutions per million (no.)				
General hospital	62.6 (49.4–83.3)	0.125	− 0.043 to 0.293	0.14
Large hospital (≥ 500 beds)	3.25 (2.40–4.18)	0.859	− 0.348 to 2.066	0.16
Hospital with long-term care beds	30.5 (25.0–45.8)	0.001	− 0.222 to 0.223	1.00
Critical care medical center	2.26 (1.87–2.83)	− 2.703	− 4.128 to − 1.279	0.001
Public health center	5.86 (3.91–8.15)	0.473	− 0.356 to 1.303	0.25
Number of beds (no.)				
Hospital beds per 10,000	138 (113–164)	− 0.085	− 0.194 to 0.024	0.12
ICU beds per 100,000	4.90 (4.05–6.46)	− 0.384	− 1.001 to 0.233	0.21
Arrival time from call to hospital (min)	38 (35–40)	− 0.118	− 0.306 to 0.070	0.21
Number per million (no.)				
Paramedics per 10,000	2.69 (2.22–3.33)	− 0.326	− 2.675 to 2.023	0.78
Medical control per million	12.0 (6.1–20.6)	0.050	− 0.108 to 0.209	0.52
Potential confounders				
Population density (no./km^2^)	266 (174–470)	− 0.001	− 0.002 to 0.001	0.39
Population of 65 years of age and older (%)	16.2 (14.5–17.2)	− 0.653	− 1.726 to 0.421	0.22
University rate	51 (46–56)	0.058	− 0.211 to 0.327	0.66
Income at capital, × 10,000 yen	321 (303–342)	− 0.054	− 0.121 to 0.012	0.10

*IQR* interquartile range, *CI* confidence interval, *ICU* intensive care unit

## Discussion

Low PCR-positive ratio, especially ≤ 4%, was associated with low COVID-19 mortality. Low PCR-positivity may imply that patients have easy access to PCR testing in the community, even if patients do not have signs or symptoms of typical pneumonia or display only mild symptoms. A greater number of critical care medical centers per million population was likewise associated with low COVID-19 mortality, whereas numbers of hospital beds, ICU beds, and other medical capacities per million population used in this analysis were not, suggesting that critical care medical centers may play an important role in reducing the risk of COVID-19 death in the community. Indeed, several reports with favorable outcomes from critical care medical centers have been reported [5–8]. They have the ability of managing veno-venous extracorporeal membrane oxygenation (ECMO) for critical COVID-19 patients [7, 8]. Thus, it is important to have not only the equipment but also the medical personnel capable of treating critical condition in COVID-19 patients, including ECMO management.

The primary limitation of this study was the ecological design. Consequently, this study only proposes the hypothesis that capacities in PCR testing and critical care medical centers may be suitable targets for enhancement.

## Conclusions

Low PCR-positive ratio, especially ≤ 4%, was associated with low COVID-19 mortality. Critical care medical centers may also play an important role in reducing the risk of COVID-19 death.
